# Reproducibility in ecology and evolution: Minimum standards for data and code

**DOI:** 10.1002/ece3.9961

**Published:** 2023-05-10

**Authors:** Gareth B. Jenkins, Andrew P. Beckerman, Céline Bellard, Ana Benítez‐López, Aaron M. Ellison, Christopher G. Foote, Andrew L. Hufton, Marcus A. Lashley, Christopher J. Lortie, Zhaoxue Ma, Allen J. Moore, Shawn R. Narum, Johan Nilsson, Bridget O'Boyle, Diogo B. Provete, Orly Razgour, Loren Rieseberg, Cynthia Riginos, Luca Santini, Benjamin Sibbett, Pedro R. Peres‐Neto

**Affiliations:** ^1^ John Wiley & Sons Ltd Oxford UK; ^2^ School of Biosciences, Ecology and Evolutionary Biology, University of Sheffield Shefield UK; ^3^ CNRS, AgroParisTech, Ecologie Systématique Evolution, Université Paris‐Saclay Orsay France; ^4^ Department of Zoology, Faculty of Sciences University of Granada Granada Spain; ^5^ Harvard University Herbaria Cambridge Massachusetts USA; ^6^ Sound Solutions for Sustainable Science Boston Massachusetts USA; ^7^ Wiley‐VCH GmbH Weinheim Germany; ^8^ Department of Wildlife Ecology and Conservation University of Florida Gainesville Florida USA; ^9^ York University Toronto Ontario Canada; ^10^ John Wiley & Sons Ltd Shanghai China; ^11^ Department of Entomology University of Georgia Athens Georgia USA; ^12^ University of Idaho Hagerman Idaho USA; ^13^ Nordic Society Oikos Lund University Lund Sweden; ^14^ Instituto de Biociencias, Universidade Federal de Mato Grosso do Sul Campo Grande Brazil; ^15^ Gothenburg Global Biodiversity Centre Goteborg Sweden; ^16^ Biosciences University of Exeter Exeter UK; ^17^ Department of Botany and Biodiversity Research Centre University of British Columbia Vancouver British Columbia Canada; ^18^ School of Biological Sciences The University of Queensland St Lucia Queensland Australia; ^19^ Department of Biology and Biotechnologies “Charles Darwin” Sapienza University of Rome Rome Italy; ^20^ Department of Biology Concordia University Montréal Quebec Canada

**Keywords:** Theorectical ecology

## Abstract

We call for journals to commit to requiring open data be archived in a format that will be simple and clear for readers to understand and use. If applied consistently, these requirements will allow contributors to be acknowledged for their work through citation of open data, and facilitate scientific progress.

## 1 WHY SHOULD RESEARCHERS SHARE DATA AND CODE?

Historically, ecologists and evolutionary biologists have had few formal incentives to share their data (Reichman et al., 2011), although informal sharing via personal communication is a long‐held practice, and there are strong practices in place around sharing and publishing molecular data. However, it is no longer seen as best practice to publish just a summary of the data linked to a scientific article within our fields. In the past decade, there has been important movement toward the standpoint that data itself must be included directly as part of research publications, as scientific progress is facilitated by accessible and transparent data.

Open science promotes and embraces more transparent and replicable research (Allen & Mehler 2019) but it is not synonymous with open data. Rather, open data, which should include code and supporting documentation, allow independent researchers to evaluate a study's methodologies and interpretations of data (Shaw et al., 2016) and use its primary data in new investigations, including data syntheses (Whitlock et al., 2010). Governments in Europe and the United States now assert that the public must have immediate access to published work and the data that support it. This assertion, when combined with increasing demand for global syntheses from policymakers (e.g., IPBES, IPCC, GBO, and UNESCO), makes it crucial that data and code (henceforth “open data”) associated with a published research project be made freely and widely accessible.

Sharing of data and application of synthesis tools allow assessment of the generality of findings (Shaw et al., 2016). Open data are a “public good,” often being funded by taxpayers, and allow researchers to apply them to future questions (Renaut et al., 2018). Additionally, data availability can generate invaluable training opportunities from reproducing previous analyses (“learning by doing”) to improving analytical skills (“learning by improving”). Finally, the benefits of publishing data and code at any time can be substantial (Lortie 2021).

For all these reasons, journals and funding bodies increasingly are requiring authors to make their data and code freely accessible (Wilkinson et al., 2016). However, there is still a great deal of variation among journals and researchers in terms of whether open data sharing should be mandated or merely encouraged and, if they are shared, whether they should be held to a minimum set of standards (Hampton et al., 2013; Herold, 2015; Whitlock et al., 2010). Too often open data are uploaded piecemeal, with no accompanying metadata or missing context on processing that happened before data deposition (Parker et al., 2016). As a result, potential for their reuse in either replicated studies, or in metanalyses that require further analytics, or their use in generating novel results or syntheses is reduced (Kelly 2019). We think that we can do better if we all follow some basic “best practices” that move our fields toward contemporary goals. Setting standards is a challenge, but one that will benefit from considering domain‐specific open data representation and needs (Poisot et al., 2019).

We call for journals to commit to requiring open data to be archived in a format that will be simple and clear for readers to understand and use. Open data should be uploaded in accordance with the FAIR (Findable, Accessible, Interoperable and Reusable data principles; https://force11.org/info/the‐fair‐data‐principles/), which are used and accepted in ecology and evolutionary biology. If applied consistently, these requirements will allow contributors to be acknowledged for their work through citation (e.g., via a DOI) of open data, and facilitate scientific progress.

## 2 WHAT DATA AND CODE SHOULD WE REQUIRE?

We call for a requirement, as a condition for publication, that authors provide all raw data and metadata, code, programming scripts, and bespoke software necessary for fully replicating any analyses that lead to inferences made in a published study. There are a number of data repositories (e.g., Dryad and Figshare) that can generate a “Private for Peer Review” link, so that data can be seen only by editor/reviewers. Key features of the data publishing framework are as follows:

1. Detailed metadata with a README file, describing relevant details about data collection, processing, analysis and presentation (e.g., Whitlock et al., 2010). See below for details and rationale.
2. Organized and clearly labeled data tables and files.
3. Clearly outlined steps for data processing as described in the associated study.
4. If bespoke scripts, analysis, or modeling methods were used, all associated programming scripts, software, and code required to run any analyses used in the study. Code functionality is the authors' responsibility, but will be additionally checked by editors provided they have the necessary resources to do so.
5. Clear and consistent file naming, avoiding long names, spaces, and special characters.
6. References to other data, where applicable. Primary data should be included, either via repository upload or in the Appendix (provide DOIs). Secondary data users should cite the original data resources and respect all the conditions of data (re)use set by the data creators or managers. See below section on ‘Data reuse’ for further detail
7. Data should be stored in an open and re‐usable format (e.g., .csv or .txt files for tables, not PDFs, and scripts in the original software format, not .txt).
8. Clearly stated license under which the data are distributed (e.g., Creative Commons).

We recognize that even open science tools such as the R programming language and Python vary between versions, instances, and operating systems. Consequently, practices associated with clean and reproducible coding should also be reviewed to ensure that reasonable documentation is provided (Filazzola & Lortie, 2022).

All of the above should be freely downloadable and preferably released immediately. On rare occasions, and with agreement from the editors after discussion with authors, an embargo period with sufficient justification may be granted. The data should be available under an accessible data usage license, and should be accessed via a permanent digital object identifier (which can be cited and points to the data in a data repository that fulfills the below criteria):

- Fully open with no barriers to access. Passworded access, even if free of charge/conditions, would not be allowed, as it can lead to complications with machine reading.
- The record is permanent after creation, and any updates, including corrections, should be clearly linked in numbered versions.

If open data are already available, redeposition is actively discouraged to avoid proliferation of duplicate datasets. If open data can be downloaded via websites that do not allow redistribution, citation of the website is permissible as long as this is accessible by anyone without conditions.

In general, the choice of repository is up to the author, but we advise a field‐specific repository. Note that some standardized types of data have specialized repositories (e.g., GenBank for sequencing data and Movebank for tracking data). Although popular, GitHub is not an acceptable repository for code, as it does not assign DOIs; in contrast, repositories such as Zenodo serve this purpose for code. GitHub's version control system, while useful in software development, is counterproductive with respect to reproducibility. Authors should check each journal's author guidelines for further information on specific repositories. If in doubt, the best policy is to contact the journal editorial office. Many data repositories also provide advice on or even facilitate data quality assurance/quality control (QA/QC).

## 3 WHY SHOULD WE SHARE METADATA?

Metadata, sometimes referred to as data about data or a data dictionary, are a “centralized repository of information about data such as meaning, relationships to other data, origin, usage, and format” (IBM Dictionary of Computing, 10th edition, 1993). They should be viewed as linked data with inherent value for reuse and discovery (Boettiger, 2019). It is widely accepted that individuals, populations, communities, and ecosystems are likely to differ even across short time scales (Powers & Hampton, 2019). Therefore, data are often strongly associated with the temporal and/or geospatial context of the sample or observation (Powers & Hampton, 2019), which makes complete and accurate metadata vital (e.g., Hoban et al., 2020; Tannenbaum et al., 2019).

Despite the recognized importance of archived metadata, records frequently do not satisfy current standards, thereby greatly diminishing the potential reuse of the associated data (Pope et al., 2015; Toczydlowski et al., 2021). Comprehensive archiving of metadata is therefore imperative to maximize the enduring value of published data.

## 4 WHAT METADATA SHOULD WE REQUIRE?

Except for four categories outlined in the “Limitations” section below, all data should be accompanied by clear metadata. Any exceptions should be clearly flagged. There are standard metadata definitions, including ecological metadata language (EML), available at https://eml.ecoinformatics.org/, and these should be used.

We acknowledge the difficulty of mandating which specific metadata are appropriate, as they depend on the nature of the study and the field. However, wherever possible, the following guidelines should be followed. If in doubt, the main criterion remains that the metadata should facilitate the full replication of any published analyses:

1. Spatiotemporal data

Crucial spatial and temporal data should be reported, including at a minimum:

- Locality identifiers and geographic coordinates in decimal degrees (unprojected latitude and longitude), or in a projected format for which the geographic projection is used as reported so as to allow reprojection. Geographic coordinates should always be associated with a measure of uncertainty.
- Time of collection that matches analytical resolution.

2 Taxonomic data

The species name should be provided in binomial format, combined with the taxonomy used (where appropriate).

3 Genetic data

Each sample should have a unique identifier that can be permanently linked to associated genetic data. For museum specimens, associated data tied to the unique identifier should include the specimen identifier, the museum voucher specimen code, the location (e.g., museum) where it is stored and any other additional information on the nature of the sample (e.g., skin/egg/skeletal structure). Identifiers in raw and processed data files should be easily cross‐referenced, for example, with look‐up tables provided whenever practicable.

4 Controlled vocabularies

If applicable, genetic metadata descriptors should be compatible with Minimum Information about any Marker Sequence (MixS, https://gensc.org/mixs/) standards.

5 README

A clear README file, specific to the metadata. If it is not clear how the data in any of the columns were collected, the README file should clarify this.

## 5 HOW SHOULD THE LINKS TO DATA AND CODE BE PRESENTED?

All links to open data associated with the paper should be presented in a Data Accessibility Statement, included at the end of the manuscript, and separate from the Acknowledgements. Any citations to datasets should be included within the references. It is expected that authors provide references and/or citations to datasets, with the journal marking them up as data citations. Data statements may vary considerably but see below for some examples:

### 5.1 Genetic data

- Raw sequence reads are deposited in the SRA (Bioproject XXXX).
- Individual genotype data are available on Dryad (XXXX).
- Unique haplotype data have been deposited in NCBI (XXXX).

### 5.2 Sample metadata

- Related metadata can be found at XXXX (including georeferences in decimal degrees and date/month/year of sampling event) and XXXX, which provides unique sample identifier tags that can be matched to both the deposited genetic data and deposited metadata (for haplotypes, individual sample identifiers and their corresponding haplotype).

### 5.3 Species distribution modeling

- Location records and environmental variables used to generate the models are made available on Dryad/Figshare (XXXX).
- Modeling procedures reported followed the ODMAP protocol (Zurell et al., 2020, Ecography 43: 1261–1277, https://doi.org/10.1111/ecog.04960).

## 6 ACCEPTABLE EXEMPTIONS

Publicly accessible sensitive research data could cause adverse effects on research participants or particular species, including discrimination of individuals, disturbances, and putting endangered species at risk of poaching and harassment (Hrynaszkiewicz et al., 2010; Lindenmayer & Scheele, 2017). Consequently, there may be times when limiting sensitive data sharing should be allowed, including:

### 6.1 Human participants

To protect the privacy of individuals, authors should obtain approval from their research ethics committee and consent from research participants before collecting and sharing data. When ethically and legally permitted, authors should seek to share aggregated or anonymized data openly with their manuscript and to share richer data through an appropriate controlled access repository.

Datasets should ideally be available upon request at minimum, whether from the authors or via a controlled access repository, and we encourage authors to design their ethical consent processes with this in mind. At the same time, we acknowledge that this ideal is not always realistic or achievable. In all cases, we encourage authors to be as transparent as possible in their data availability statement. For data that are available upon request, authors should describe the application process, conditions for access, and the controlling data accession committee, if relevant. If any datasets cannot be shared, this should be clearly stated with a succinct explanation.

Openly shared datasets should not include direct identifiers (e.g., names, email addresses, etc.) and should avoid multiple indirect identifiers (e.g., gender, age, and occupation). For more tips on de‐identifying and sharing sensitive research data, see Hrynaszkiewicz et al. (2010). Data sharing should follow all requirements imposed by the relevant ethics review entity and all laws for the regions in which the data are collected, housed, or published.

We encourage authors to use the centralized, community‐maintained controlled‐access repositories that are appropriate for their data. Exemplars include the European Genome‐phenome Archive (EGA, https://ega‐archive.org/) and the Database of Genotypes and Phenotypes (dbGaP) (https://www.ncbi.nlm.nih.gov/gap/) for genetic data, or the Harvard Dataverse (https://dataverse.harvard.edu/) for other types of data. Many other options exist, and authors should take care to choose a repository that is appropriate for their ethical and legal context.

### 6.2 Endangered species

To protect endangered or threatened species from further decline, authors should evaluate the risk level and threats of individual species using the IUCN Red List (https://www.iucnredlist.org/) and assess whether sharing the data could cause harm to the species before sharing the data. Ethical and reasonable practices that protect species but enable some levels of transparency should be given due consideration. A Red List threatened category does not automatically qualify data for a data‐sharing exemption, as a species may be threatened by factors that would not be exacerbated by releasing locality data (e.g., pollution, habitat loss, etc.), or only in part of their geographic range. Examples that may qualify are species that are locally threatened because of poaching, collection, or harassment. In the majority of these cases, decreasing the resolution (precision) of the locality data may be sufficient to avoid harm. Alternatively, the precise location information of endangered species should be anonymized (Chapman, 2020; Lindenmayer & Scheele, 2017) while preserving the ability to reproduce the analyses. For example, in some multispecies analyses, the analyses may be fully reproducible without knowledge of individual species names or precise geographic locations, as long as environmental covariates are provided.

### 6.3 Long‐term research data

Authors are often allowed no more than a one‐year embargo period before their persistent identifier goes live. If authors require a longer embargo period, this may be granted with sufficient reason (Whitlock et al., 2016), at the discretion of the editors. Automatic release after the embargo period is required. Documentation from funders is welcome to support any embargo requests.

### 6.4 Indigenous recognition

If data collection has taken place on territory which is subject to legal or traditional recognition of indigenous traditions and management practices, or with conditional cooperation from indigenous communities, authors and journals should clearly acknowledge this. An exemption may be permitted in accordance with agreements such as the Nagoya Protocol (e.g., Marden et al., 2021).

## 7 REUSE OF OPEN DATA

Authors should clearly distinguish between open data that already are publicly available, and open data that are proprietary. Most open data available in public repositories can be reused without permission, but the specific license should be checked in each instance. In cases where open data are not reusable without permission, users should cite the original data resources and respect all the conditions of (re)use set by the original researchers or creators. Authors should be looking from the earliest possible stage to work with proprietary owners to release the data on publication. In cases where proprietary open data are not owned by any of the authors and permission to archive the data is not granted, evidence of any refusals (e.g., email exchanges) by third parties must be sent to the editorial office. For proprietary open data that the authors do not have permission to use, they must seek permission to archive them. In general, there should be an expectation that all data creators or compilers will be contacted to be made aware of the reuse of the data.

## AUTHOR CONTRIBUTIONS

**Gareth B. Jenkins:** Conceptualization (lead); writing – original draft (equal); writing – review and editing (equal). **Andrew P. Beckerman:** Writing – original draft (equal); writing – review and editing (equal). **Céline Bellard:** Writing – original draft (equal); writing – review and editing (equal). **Ana Benítez López:** Writing – original draft (equal); writing – review and editing (equal). **Aaron M. Ellison:** Writing – original draft (equal); writing – review and editing (equal). **Christopher G. Foote:** Writing – original draft (equal); writing – review and editing (equal). **Andrew L. Hufton:** Writing – original draft (equal); writing – review and editing (equal). **Marcus A. Lashley:** Writing – original draft (equal); writing – review and editing (equal). **Christopher J. Lortie:** Writing – original draft (equal); writing – review and editing (equal). **Zhaoxue Ma:** Writing – original draft (equal); writing – review and editing (equal). **Allen J. Moore:** Writing – original draft (equal); writing – review and editing (equal). **Shawn R. Narum:** Writing – original draft (equal); writing – review and editing (equal). **Johan Nilsson:** Writing – original draft (equal); writing – review and editing (equal). **Bridget O'Boyle:** Writing ‐ original draft (equal); writing ‐ review and editing (equal). **Diogo B. Provete:** Writing – original draft (equal); writing – review and editing (equal). **Orly Razgour:** Writing – original draft (equal); writing – review and editing (equal). **Loren Rieseberg:** Writing – original draft (equal); writing – review and editing (equal). **Cynthia Riginos:** Writing – original draft (equal); writing – review and editing (equal). **Luca Santini:** Writing – original draft (equal); writing – review and editing (equal). **Benjamin Sibbett:** Writing ‐ original draft (equal); writing ‐ review and editing (equal). **Pedro R. Peres‐Neto:** Writing – original draft (equal); writing – review and editing (equal).

## Data Availability

No data or code were generated for this manuscript.
